# Molecular characterization of MAP9 in the photoreceptor sensory cilia as a modifier in canine *RPGRIP1*-associated cone-rod dystrophy

**DOI:** 10.3389/fncel.2023.1226603

**Published:** 2023-08-15

**Authors:** Kei Takahashi, Jennifer C. Kwok, Yu Sato, Gustavo D. Aguirre, Keiko Miyadera

**Affiliations:** Division of Experimental Retinal Therapies, Department of Clinical Sciences & Advanced Medicine, School of Veterinary Medicine, University of Pennsylvania, Philadelphia, PA, United States

**Keywords:** animal model, canine, inherited retinal diseases, MAP9, microtubule-binding protein, photoreceptor, primary cilia, RPGRIP1

## Abstract

Photoreceptors possess a highly specialized primary cilium containing expanded ciliary membrane discs called the outer segment. The photoreceptor cilium is essential for the maintenance of the outer segment, and pathogenic variants in more than 50 cilia-related genes have been identified as causing non-syndromic inherited retinal diseases in patients. The retinitis pigmentosa GTPase regulator interacting protein 1 (RPGRIP1) is a structural protein localized to the photoreceptor cilium and biallelic *RPGRIP1* variants have been associated with non-syndromic human inherited retinal diseases. In a canine cone-rod dystrophy model, a naturally occurring 44-bp exonic insertion in *RPGRIP1* (*RPGRIP1*^*ins*44/*ins*44^) is the primary disease locus while an additional homozygous variant in *MAP9* (microtubule associated protein 9) (*MAP9^aff/aff^*) acts as a modifier associated with early disease onset. MAP9 was originally identified as a microtubule-binding protein stabilizing microtubule structure during both mitosis and interphase in human cell lines. However, the roles of MAP9 in primary cilia, including photoreceptor neurosensory cilia, have not been well understood. Hence, we characterized the pathogenic phenotypes associated with homozygous *MAP9* variant, and investigated the molecular function of MAP9 in primary cilia using the *RPGRIP1*-associated oligogenic canine cone-rod dystrophy model as well as cultured cells. Both functionally and structurally, the *RPGRIP1*^*ins*44/*ins*44^ *MAP9^aff/aff^* retina exhibited progressive cone photoreceptor degeneration starting earlier than the retina affected by *RPGRIP1*^*ins*44/*ins*44^ alone. Based on immunostaining of canine retinal sections and cultured cells, we found that MAP9 is prominently localized in the basal body of primary cilia and played an important role in maintaining the structure of ciliary microtubule axoneme. These findings suggest that the affected MAP9, together with mutant RPGRIP1, is deprived of critical roles in cilia organization and maintenance resulting in altered cilia structure and function giving rise to early onset and accelerated disease progression in the *RPGRIP1*^*ins*44/*ins*44^ *MAP9^aff/aff^* double homozygote cone-rod dystrophy canine model.

## Introduction

In vertebrates, photoreceptors are specialized neurons located in the outermost retinal layer and are responsible for the detection of light and its conversion into electrical signals, processes known as photoreception and phototransduction. There are two types of photoreceptor cells: rods and cones. Rod photoreceptors are responsible for dim light vision, while cone photoreceptors are involved in visual acuity and color perception. Both types of photoreceptors possess the outer segment (OS) which is a highly specialized and optimized primary cilium containing expanded ciliary membrane discs that are enriched in rhodopsin or cone opsin. This structure is anchored to the inner segment (IS) by a ciliary microtubule (MT) axoneme extending from the connecting cilium (CC), which possesses many features resembling the transition zone of the primary cilia (Barnes et al., 2021; Wensel et al., 2021). Since the OS lacks the machinery for protein synthesis and metabolism, these cellar functions take place in the IS and nutrients and other molecules are transported to the OS through the CC. Thus, the CC is essential for the maintenance of the OS function and structure, and defects in cilia-related genes are directly associated with photoreceptor degeneration. To date, pathogenic variants in more than 50 cilia-related genes have been identified as causing non-syndromic inherited retinal diseases (IRDs) in human patients (Sánchez-Bellver et al., 2021).

The retinitis pigmentosa GTPase regulator (RPGR) interacting protein 1 (RPGRIP1) is a structural protein localized to the photoreceptor CC and modulates the localization of several cilia-associated proteins, such as RPGR (Hong et al., 2001), nephrocystin-4 (NPHP4) (Roepman et al., 2005), centrosomal protein 290 (CEP290) (Chang et al., 2006), serologically defined colon cancer antigen-8 (SDCCAG8) (Patil et al., 2012), and spermatogenesis-associated protein-7 (SPATA7) (Eblimit et al., 2015). Biallelic *RPGRIP1* variants have been associated with non-syndromic human IRDs, including retinitis pigmentosa (RP) (Booij et al., 2005), cone-rod dystrophy (CRD) (Hameed et al., 2003), and Leber congenital amaurosis (LCA) type 6 (Dryja et al., 2001). Notably, these human *RPGRIP1*-associated IRDs exhibited extensive clinical and phenotypic heterogeneity (Beryozkin et al., 2021; Mao et al., 2021).

Heterogeneous IRDs have also been recognized in over 100 breeds of dogs in which many forms of IRDs share genetic and pathologic features with their human counterparts (Miyadera et al., 2012a). In a closed breeding colony of Miniature Longhaired Dachshunds (MLHD), a naturally-occurring homozygous 44-bp exonic insertion in *RPGRIP1* (*RPGRIP1*^*ins*44/*ins*44^) has been associated with canine cone-rod dystrophy 1 (cord1) (Mellersh et al., 2006). This genetic variant was predicted to cause a frameshift and premature termination of RPGRIP1, and there was a complete genotype-phenotype correlation in the initially characterized research colony. However, further study with the broader population of pet MLHD has shown extensive phenotypic variation in age of onset and disease progression among dogs sharing the identical *RPGRIP1* variant (Miyadera et al., 2009). To search for a genetic factor controlling the variable *RPGRIP1*^*ins*44/*ins*44^ phenotype, a genome-wide association study was conducted using canine SNP microarray. Genotyping data from 83 *RPGRIP1*^*ins*44/*ins*44^ MLHDs revealed a single locus on canine chromosome 15 associated with the early onset of cord1 (Miyadera et al., 2012b). Finally, target-enriched sequencing of this locus identified an approximately 22 kb deletion in the gene encoding microtubule associated protein 9 (MAP9) as a modifier associated with an early disease onset (Forman et al., 2016).

MAP9, also known as ASter-Associated Protein (ASAP), was originally identified as a MT-binding protein stabilizing MT structure during both mitosis and interphase in human cell lines (Saffin et al., 2005). This protein can directly interact with MTs via the C-terminal domain and depletion of MAP9 leads to delay of mitotic progression and failure of cytokinesis. Thus, associations between MAP9 dysfunction and the development of tumor cells has been reported in several types of carcinomas (Liu et al., 2018; Wang et al., 2020; Zhang et al., 2020). There are also several evidences that implicate MAP9 in the formation and function of primary cilia; specific expression of the *C. elegans* MAP9 ortholog MAPH-9 in ciliated sensory neurons (Jensen et al., 2016; Magescas et al., 2021); *map9* depletion inducing developmental abnormalities related to cilia-dependent signaling pathways in zebrafish (Fontenille et al., 2014); and localization of MAP9 in the photoreceptor CC region of canine retina (Das et al., 2017). Despite these reports suggesting the involvement of MAP9 in MT organization and ciliary function, the role of MAP9 in the primary cilia, including photoreceptor neurosensory cilia, has not been adequately established fully.

Hence, in this study, we characterized the pathogenic phenotypes associated with the homozygous *MAP9* variant, and investigated the molecular function of MAP9 in primary cilia using the *RPGRIP1*-associated oligogenic canine CRD model.

## Materials and methods

### Animals

The origin of the *RPGRIP1* variant canine research colony has been described previously (Kuznetsova et al., 2012; Das et al., 2017). The colony was maintained at the Retinal Disease Studies Facility (RDSF) of the University of Pennsylvania and housed under identical conditions of diet, medications, vaccinations, and ambient illumination, with cyclic 12 h ON-12 h OFF. All the canines enrolled in this study were typed for the known *RPGRIP1* and *MAP9* genetic variants. The 44 bp insertion in *RPGRIP1* and the ∼22 kb deletion in *MAP9* were typed by a modified PCR-gel electrophoresis protocol based on previous reports (Mellersh et al., 2006; Forman et al., 2016). The dogs were categorized into three groups: control, *RPGRIP1* single affected (*RPGRIP1*^*ins*44/*ins*44^ with *MAP9^wt/wt^* or *MAP9^wt/aff^*), and *RPGRIP1/MAP9* double affected (*RPGRIP1^*ins*44/*ins*44^ MAP9*^aff/aff^**), according to the modifier genotypes (Table 1). Recently, we mapped a novel modifier locus as the third cord1 locus (L3) which appear to exacerbate the cone photoreceptor dysfunction of *RPGRIP1*-associated cord1 (Ripolles-Garcia et al., 2023). In the current study, only the animals heterozygous and hence unaffected by the recessive L3 locus, were used to investigate the unique pathological impacts of the *MAP9* variant (Table 1).

**TABLE 1:** Genetic subpopulations and their grouping in the current study.

Dog ID	Sex	Genotypes / haplotype			ERG	OCT	Histological analysis	Skin biopsy	References
		*RPGRIP1*	*MAP9*	L3					
**Control**									
R83	F	wt/ins44	wt/aff	wt/aff		✓*			Ripolles-Garcia et al., 2023
R96	M	wt/ins44	wt/aff	wt/aff	✓		✓ (17 months)	✓	Current study
R98	F	wt/ins44	wt/aff	wt/aff	✓				
***RPGRIP1* ^*ins*44/*ins*44^**									
R42	M	ins44/ins44	wt/aff	wt/aff			✓ (5 years 9 months)		Current study
R95	M	ins44/ins44	wt/wt	wt/aff	✓				
R99	M	ins44/ins44	wt/aff	wt/aff	✓	✓	✓ (17 months)	✓	
***RPGRIP1*^*ins*44/*ins*44^ *MAP9*^*aff/aff*^**									
R67	F	ins44/ins44	aff/aff	wt/aff			✓ (6 years)		Current study
R94	M	ins44/ins44	aff/aff	wt/aff	✓	✓	✓ (17 months)	✓	

aff, affected; ERG, electroretinograms; ins44, 44-base pair insertion; OCT, optical coherence tomography.

**✓**, used for each examination.

*, control canine OCT data was derived from the previous paper.

### Ophthalmic examination

Routine ophthalmic examinations including slit-lamp biomicroscopy, tonometry, indirect ophthalmoscopy, and fundus photography were performed for all canines at 1 through 17 months of age. Wide-field color fundus photographs were taken using a RetCam^®^ Shuttle retinal camera (Natus Medical Inc., Pleasanton, CA, USA) with a 130-degree field-of-view lens.

### Electroretinograms (ERG)

ERG recordings were performed under scotopic and photopic stimuli as previously described (Aguirre et al., 2021) at 1 through 17 months of age. Full-field flash ERGs were recorded in both eyes using a custom-built Ganzfeld dome fitted with light-emitting diode stimuli from a ColorDome stimulator (Diagnosys LLC, Lowell, MA, USA). Amplitudes of recorded scotopic (rod) b-wave and photopic 29.4-Hz flicker (cone) were measured using the ERG software (Espion V6, Diagnosys, Lowel, MA, USA). All ERG data were obtained from the littermates (Dog ID: R94-R99).

### Spectral domain optical coherence tomography (SD-OCT)

SD-OCT imaging was carried out under general anesthesia using Spectralis^®^ HRA + sdOCT (Heidelberg Engineering, Heidelberg, Germany) as previously described (Das et al., 2017; Ripolles-Garcia et al., 2023). Outer nuclear layer (ONL) thickness was measured with single, high-resolution 30° longitudinal b-scans from the optic nerve head to the peripheral retina in both superior and inferior meridians for each canine eye. Measurements were acquired using HEYEX software (Heidelberg Engineering) and segmented manually every 500 μm from the optic nerve head. For each dog at a given time point, the ONL thickness measurements acquired from each eye were averaged to represent linear graphs across the vertical meridian.

### Immunohistochemistry (IHC)

Preparation and processing of tissues for IHC has been detailed previously (Aguirre et al., 2021). For detection of proteins related to photoreceptor cilia, retinal sections were treated with Antigen Retriever regent (Sigma-Aldrich, St. Louis, MO, USA, cat# R2283) for 1 min prior to blocking at room temperature. In all cases, retinal cryosections were incubated overnight at 4°C with commercial or custom-made primary antibodies (Supplementary Table 1) in blocking buffer (4.5% cold fish gelatin, 0.1% sodium azide, 5% bovine serum albumin (BSA), and 0.25% Triton X-100 in phosphate-buffered saline (PBS)). Antigen-antibody complexes were visualized with fluorochrome-labelled secondary antibodies (1:1,000; Alexa Fluor Dyes, Invitrogen, Carlsbad, CA, USA) and then counterstained using Hoechst 33342 (1:1,000; Thermo Fisher Scientific, Waltham, MA, USA, cat# 62249). The stained sections were photographed using SP5-II confocal microscope (Leica SP5-II; Leica Microsystems, Wetzlar, German) with a 100 × objective. Digital images were processed using the Leica Application Suite X (Leica Microsystems). The thickness of each retinal layer and the number of cone photoreceptors (hCAR^+^ cells) were measured in a 200 μm square within the 1,000–2,000 μm range from the optic disc using the ImageJ software (National Institutes of Health, Bethesda, MD, USA). Quantitative data were compared among the littermates.

### The human protein atlas (HPA) database analysis

The HPA database provides information on transcript expression levels summarized per gene in 79 human cell types.^1^ The single cell RNA-seq dataset was obtained from published studies based on healthy human tissues and meta-analysis on 30 single cell databases was performed. In this meta-analysis, normalized transcript expression values, denoted nTPM, were calculated for each gene in every sample to combine the 30 datasets into consensus transcript expression levels. Expression levels of *RPGRIP1* and *MAP9* transcripts between different cell types were compared using the dataset derived from this database.

### Cell cultures

661W cells (Tan et al., 2004), an immortalized cell line derived from murine retinal tumor and expressing cone photoreceptor featurese, were obtained from Dr. Muayyad R. Al-Ubaidi (Department of Biomedical Engineering, University of Houston, Houston, TX, USA). The 661W cells were cultured in Dulbecco’s modified Eagle’s medium (DMEM; Thermo Fisher Scientific, cat# 11885084) containing 10% fetal bovine serum (FBS; Sigma-Aldrich, cat# F2442) and 1% Antibiotic-Antimycotic regent (Thermo Fisher Scientific, cat# 15240062).

To generate primary canine skin fibroblasts (CSFs), skin biopsies were collected from each canine genotype and washed in 70% ethanol followed by rinsing with PBS containing 2% Antibiotic-Antimycotic. The tissues were minced with sterile razor blade and incubated in 5 U/mL Dispase (STEMCELL Technology, Vancouver, Canada, cat# 07913) + 1 mg/mL Collagenase type IV (STEMCELL Technology, cat# 07426) for 3 h at 37°C. After filtering with 100 μm cell strainer, the cell suspension was centrifuged for 8 min at 300 × *g*. The cells were collected and cultured in DMEM/F-12 (Thermo Fisher Scientific, cat# 11320033) containing 10% FBS (Sigma- Aldrich), 1% Antibiotic-Antimycotic (Thermo Fisher Scientific), 1% L-Glutamine (Thermo Fisher Scientific, cat# 25030081), 1% Sodium Pyruvate (Thermo Fisher Scientific, cat# 11360070), and 1% MEM Non-essential Amino Acids (Thermo Fisher Scientific, cat# 11140050). The cells were cultured in a humidified atmosphere of 95% air and 5% CO_2_ at 37°C, and passaged by trypsinization every 2 days.

### Immunocytochemistry (ICC)

The cultured cells were fixed in 4% paraformaldehyde (Santa Cruz Biotechnology, Dallas, TX, USA, cat# sc-281692) at room temperature for 10 min and then washed with D-PBS (Corning, Corning, NY, USA, cat# 21-030-CV). The cells were incubated overnight with primary antibodies (Supplementary Table 1) at 4°C following incubation in blocking buffer (4.5% cold fish gelatin, 0.1% sodium azide, 5% BSA, and 0.25% Triton X-100 in PBS) for 1 h at room temperature. The samples were stained with Alexa Fluor Dye-conjugated secondary antibodies (1:1,000; Invitrogen) and then counterstained with Hoechst 33342 (1:1,000; Thermo Fisher Scientific). The fluorochrome-labeled proteins were detected using Leica SP5-II confocal microscope (Leica Microsystems) with a 100 × objective and a fluorescence microscope (Axioplan; Carl Zeiss Meditec GmbH, Oberkochen, Germany) with a 40 × objective.

### Examination of ciliogenesis rate and ciliary length

The cells were seeded (661W, 4 × 10^4^ per mL; CSFs, 8 × 10^4^ per mL) on sterile glass coverslips in 10% FBS containing media. After 24 h of seeding, the culture media was changed to serum-free media, and cells were incubated for a further 48 h to induce ciliogenesis. The cells were fixed with 4% PFA and then the ciliary axonemes were labeled through ICC with anti-acetylated α-tubulin (AcTub) antibodies. Two-dimensional images were obtained using Axioplan microscope (Carl Zeiss Meditec GmbH) with a 40x objective lens. The number of ciliated cells and the length of ciliary axoneme were manually obtained using ImageJ (National Institutes of Health, Bethesda, MD, USA). Three-well replicates were prepared for each experimental group and 2-3 images were taken for each well. Dunn’s multiple comparisons test was performed following a significant Kruskal–Wallis test. A *P* value of < 0.05 was considered statistically significant. All statistical analyses were performed using GraphPad Prism version 9.4.1 (GraphPad software; San Diego, CA, USA).

### Cloning of canine cDNA into expression vectors and cell transfection

Retinal RNA was extracted from *MAP9* wild type (*MAP9*^*wt*/*wt*^) and affected (*MAP9*^*aff*/*aff*^) canine retinas. Full-length *MAP9* transcripts (MAP9^wt^ and MAP9^aff^) were each amplified by RT-PCR and cloned into pKMyc mammalian expression vector (Addgene, Cambridge, MA) to generate N-terminal Myc-tagged mutant- and WT-*MAP9* constructs. 661W cells were seeded at 2 × 10^4^ per mL on sterile glass coverslips in media containing 10% FBS, and after 24 h of culturing, the recombinant expression vectors were transfected to the cells using Lipofectamine 3000 (Invitrogen) in Antibiotic-Antimycotic free culture medium. After 48 h of transfection, ciliogenesis was induced by replacing the old culture media containing 10% FBS with serum-free culture media and cells were incubated for additional 48 h before harvesting to promote primary cilia formation.

## Results

### Early onset and progression of retinal degeneration in *RPGRIP1/MAP9* double affected canine

**FIGURE 1:**
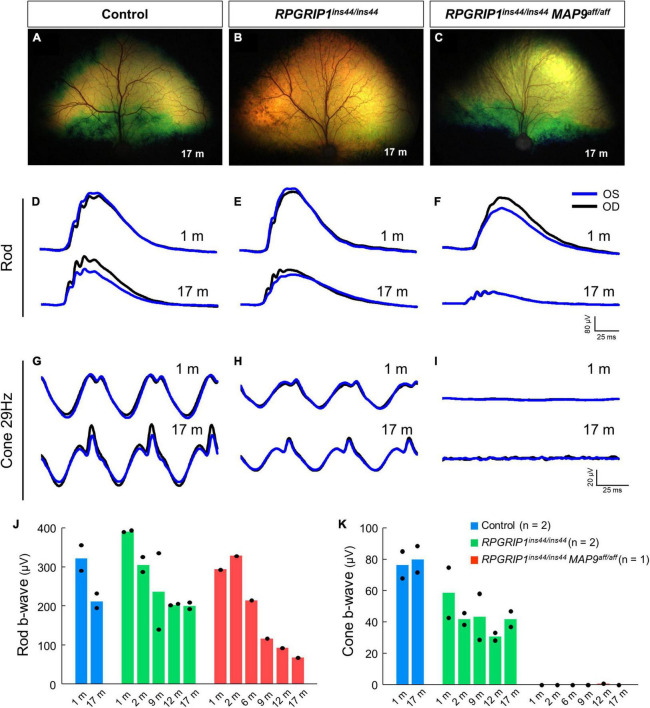
Rod and cone ERGs in the early disease stage of *RPGRIP1*-associated CRD. Fundus photographs of control **(A)**, *RPGRIP1* single affected **(B)**, and *RPGRIP1/MAP9* double affected **(C)** dogs at 17 months of age. **(D–I)** Scotopic and photopic ERG traces of representative canines of each genotype group. OD, right eye; OS, left eye. **(J,K)** Responses to full-field ERGs represented by rod **(J)** and cone 29 Hz **(K)** b-wave amplitudes (μV) from 1 to 17 months of age are displayed per age and group.

**FIGURE 2:**
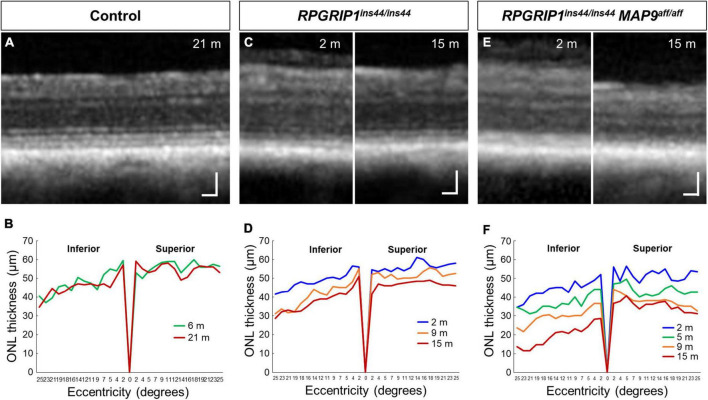
Longitudinal evaluation of ONL thickness in *RPGRIP1*-associated CRD retinas. Representative SD-OCT images of control **(A)**, *RPGRIP1* single affected **(C)**, and *RPGRIP1/MAP9* double affected **(E)** retinas. Scale bar, 50 μm. Graphs of ONL thickness (μm) measured on SD-OCT along the superior–inferior meridians of control **(B)** and *RPGRIP1* single affected **(D)** retina. The *RPGRIP1/MAP9* double affected eye shows the rapid and severe ONL thinning **(F)**.

To examine the impact of *MAP9* homozygous variant on the *RPGRIP1* phenotype at the early stage of disease, functional and structural evaluations were performed on control, *RPGRIP1* single affected and *RPGRIP1*/*MAP9* double affected retinas up to 17 months of age (Table 1). To specifically evaluate the effect of the *MAP9^aff/aff^* modifier, both the single and double affected *RPGRIP1* mutants were confirmed to be unaffected (heterozygous) by a recently identified additional modifier L3 (Ripolles-Garcia et al., 2023). Ophthalmoscopically, there were no obvious signs of retinal degeneration in the control or single affected retina up to 17 months of age (Figures 1A, B). In contrast, the double affected retina showed mild to moderate retinal vascular attenuation and tapetal hyper-reflectivity consistent with retinal atrophy by 17 months of age (Figure 1C). Rod-derived ERG response declined precipitously in the double affected while the single affected animal showed a milder progression over time (Figures 1D–F, J). Cone-derived ERG response was non-recordable in the double affected at all time points, while that of the single affected was mildly reduced and relatively stable through the 17-month period (Figures 1G–I, K). SD-OCT in the single and double affected dogs found limited ONL thinning as early as 2 months of age in the double affected only. Over time, there was rapid progression of ONL thinning in the double affected, while the single affected showed a modest progression (Figures 2A–F). These findings suggest that *MAP9^aff/aff^* accelerates the onset and progression of cone-led retinal degeneration of *RPGRIP1*-associated CRD.

### Histological disruption of photoreceptor cells at young ages in *RPGRIP1/MAP9* double affected canines

**FIGURE 3:**
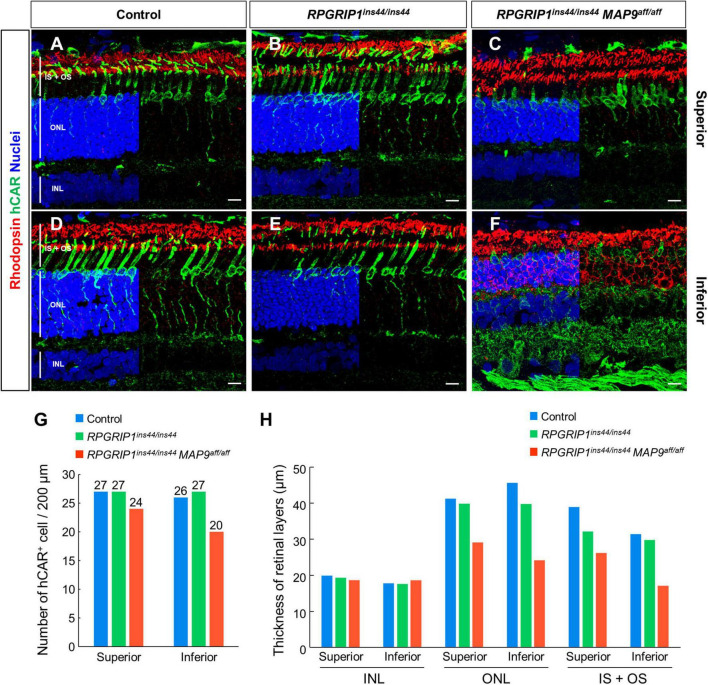
Immunohistochemical examination of photoreceptor degeneration in *RPGRIP1*-associated CRD retinas. **(A–F)** IHC on 17 month-old canine retinal cryosections with anti-hCAR (green) and rhodopsin (red) antibodies. The upper and lower panels show superior and inferior retina, respectively. Nuclei were stained with Hoechst 33342 (blue). The number of hCAR^+^ cells **(G)** and the thickness of each retinal layer **(H)** were quantitated in a 200 μm square within the 1,000–2,000 μm range from the optic disc. INL, inner nuclear layer; IS + OS, inner segment + outer segment; ONL, outer nuclear layer. Scale bar, 10 μm.

To observe the histological impact of modifier variants on photoreceptor degeneration, we performed IHC on canine retinal cryosections against rhodopsin, the most abundant photopigment in the rod OS (Hofmann and Lamb, 2023), and human cone arrestin (hCAR), which allows identifying cones even after the loss of OS and IS (Eandi et al., 2016). At 17 months of age, the photoreceptor IS + OS structure in *RPGRIP1* single affected retina was well preserved compared to those in control retina (Figures 3A, B, D, E). In contrast, the *RPGRIP1*/*MAP9* double affected retina showed cone OS loss, shortened IS, and reduced number of cone cells at the same age (Figures 3C, F, G). Consistent with the OCT imaging in Figure 2, retinal degeneration was more severe in the inferior vs. the superior quadrant in the double affected eye, showing atrophy of the rod OS, rhodopsin mislocalization in the ONL, and more pronounced shortening of the cone IS and cone OS loss in the inferior retina (Figures 3C, F). By 6 years of age, the photoreceptor degeneration in the *RPGRIP1*/*MAP9* double affected canine retina was further exacerbated in the inferior and to a lesser extent the inferior quadrant (Supplementary Figure 1). The thickness of photoreceptor layer spanning the ONL and the IS + OS was notably reduced in the double affected retina, while the inner nuclear layer (INL), the nucleus of secondary neurons, was preserved at 17 months of age (Figure 3H). These results indicate that the homozygous *MAP9* variant accelerates the progression of cone-led photoreceptor degeneration caused by *RPGRIP1*^*ins*44/*ins*44^.

### Expression and localization of endogenous RPGRIP1 and MAP9 in canine retina

To assess the expression patterns of MAP9 and RPGRIP1 *in vivo*, we used first the meta-analytic data on human single cell RNA-seq from the HPA database to identify cell types with higher levels of *RPGRIP1* or *MAP9* transcript expression. While *MAP9* transcripts are expressed in a wide range of cell types, the expression level was particularly high in “ciliated cells”, including photoreceptors (Supplementary Figure 2A and Supplementary Table 2). *RPGRIP1* transcripts were highly expressed specifically on photoreceptors and in spermatids, both of which possess a highly specialized cilia, in man (Supplementary Figure 2B and Supplementary Table 2). These results suggest that MAP9 and RPGRIP1 have specific roles in ciliated cells, especially photoreceptors.

**FIGURE 4:**
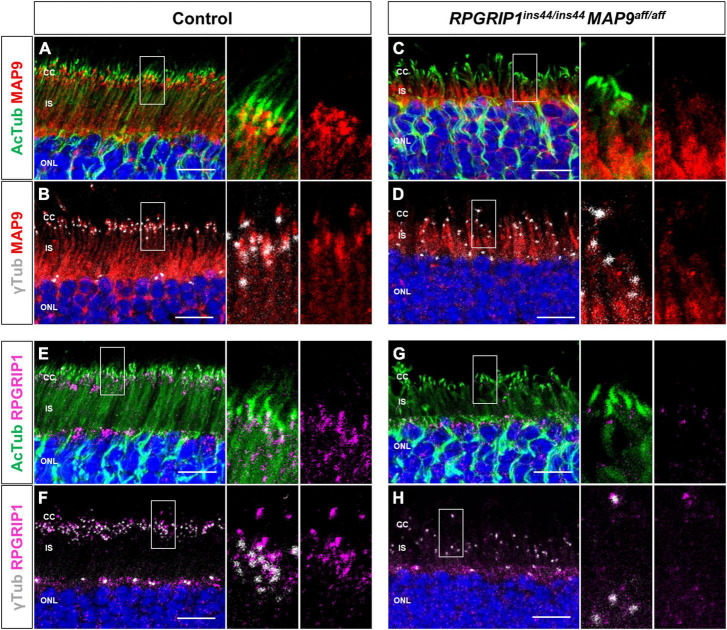
Localization of endogenous RPGRIP1 and MAP9 in canine photoreceptors. Control and *RPGRIP1/MAP9* double affected canine retinas are co-immunolabeled with the ciliary MT axonemes (AcTub, green) or the basal bodies (γ-tubulin, gray) along with either MAP9 (red) or RPGRIP1 (magenta). Control canine retina exhibits specific MAP9 signals at the base of the photoreceptor cilia **(A)**, partially overlapping with γ-tubulin signals **(B)**. MAP9 was labeled with commercially available primary antibody (Proteintech, Cat# 26078-1-AP). Strong endogenous RPGRIP1 signals were also detected at the basal part of cilium, while they merged with AcTub signals partially **(E)**, and not with γ-tubulin signals **(F)**. In the *RPGRIP1/MAP9* double affected retina, the signals of both proteins were attenuated at the base of cilia **(C,D,G,H)**. Nuclei were visualized with Hoechst 33342 (blue). CC, connecting cilium; IS, inner segment; ONL, outer nuclear layer. Scale bar, 10 μm.

Based on the above studies, we investigated the intracellular localization of endogenous MAP9 and RPGRIP1 proteins in photoreceptors using canine retinal cryosections. We have previously suggested that MAP9 localizes adjacent to the photoreceptor OS (Das et al., 2017). Here, IHC was performed against AcTub and γ-tubulin as markers of ciliary MT axonemes and basal bodies (BB), respectively, to investigate the expression pattern of each protein in the photoreceptor cilium. In the control retina, strong endogenous MAP9 signals were detected at the base of the photoreceptor cilia, partially overlapping with AcTub and γ-tubulin signals (Figures 4A, B and Supplementary Figure 3). Distinct RPGRIP1 signals were also observed at the basal part of cilium, while they merged with AcTub signals partially, but not with γ-tubulin signals (Figures 4E, F). These data suggest that MAP9 and RPGRIP1 are localized primarily between the BB to the CC, respectively. In the *RPGRIP1*/*MAP9* double affected retina with progressive disease, the signals of both proteins were attenuated at the base of cilia (Figures 4C, D, G, H).

### Extension of endogenous MAP9 into the ciliary axoneme in cultured cells

**FIGURE 5:**
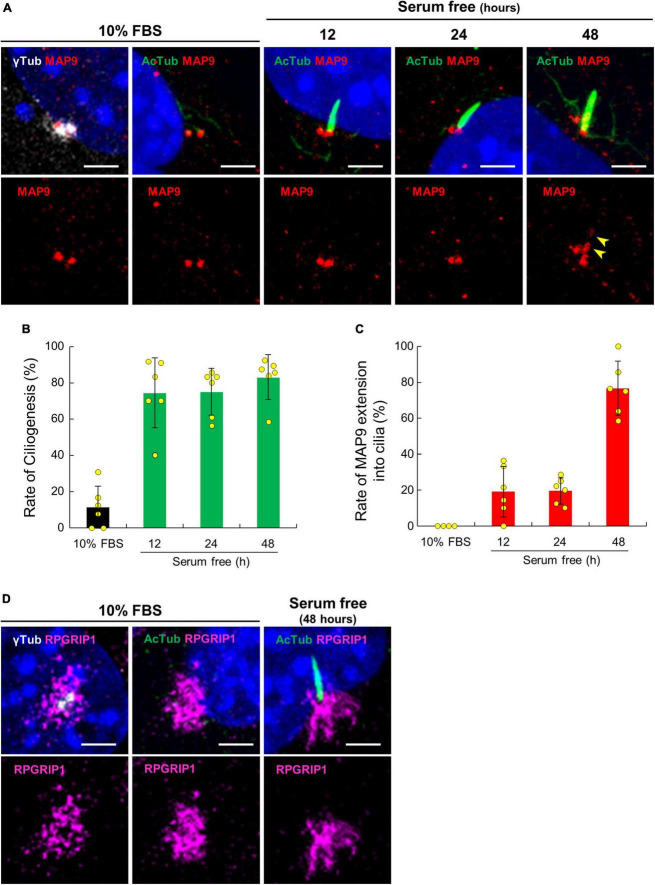
Ciliary localization of endogenous MAP9 and RPGRIP1 in 661w cells. **(A)** 661W cells cultured with or without FBS are co-immunolabeled with anti-AcTub (green) or γ-tubulin (gray) antibodies along with anti-MAP9 antibody (red). *Upper* panels show labels by two antibodies and nuclei; *Lower* panels show only the MAP9 signals. The yellow arrowheads show MAP9 signals extended into the ciliary axoneme. Histograms show the percentages of ciliated cells **(B)** and cells with MAP9 extension into the ciliary axoneme **(C)**. Bars represent mean ± SD. **(D)** Endogenous RPGRIP1 signals in 661W cells were visualized with anti-RPGRIP1 antibodies (magenta). *Upper* panels show labels by two antibodies and nuclei; *Lower* panels show only the RPGRIP1 signals. Nuclei were stained with Hoechst 33342 (blue). Scale bar, 5 μm.

To validate the localization of MAP9 and RPGRIP1 in primary cilia, we performed ICC with a cell culture model using mouse-derived immortalized retinal tumor cell line, 661W (Tan et al., 2004; Wheway et al., 2019). Under the condition with 10% serum, endogenous MAP9 signals were localized as two juxtaposed puncta and clearly co-localized with γ-tubulin (Figure 5A). By 48 h of serum starvation, ciliary axonemes were identified in approximately 80% of 661W cells, extending from one of the two MAP9-positive puncta (Figures 5A, B). Notably, endogenous MAP9 signals extended into the ciliary axoneme in approximately 80% of ciliated cells after 48 h of serum starvation, even though MAP9 signal remained primarily in the base of the cilia in the early phase of ciliogenesis (Figures 5A, C). These findings suggest that endogenous MAP9 is abundantly localized in the centrosomes and the ciliary BB, and extends to the ciliary axonemes with cilia maturation. In contrast, RPGRIP1 did not co-localize with either γ-tubulin or AcTub but was found around these proteins (Figure 5D).

### Impaired ciliary axoneme with *MAP9* modifier variant in the primary cilium

**FIGURE 6:**
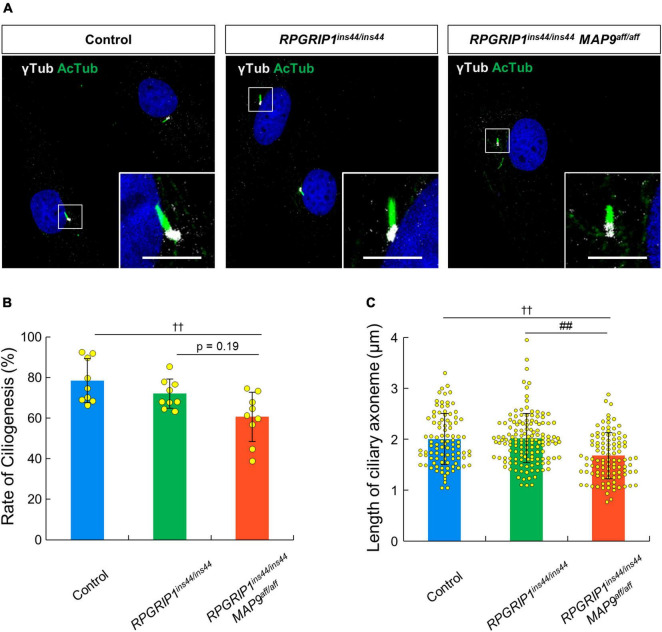
*MAP9^aff/aff^* affects the rate of ciliogenesis and the length of primary cilia in canine skin fibroblasts. **(A)** Representative ICC images of control, *RPGRIP1* single affected, and *RPGRIP1/MAP9* double affected CSFs. The ciliary MT axonemes and the basal bodies were visualized with anti-AcTub (green) and γ-tubulin (gray) antibodies. Scale bar, 5 μm. Histograms show the rate of ciliogenesis [**(B)** Number of wells = 3, Number of images = 9] and the length of ciliary axoneme [**(C)** Number of wells = 3, Number of cells > 90] of each genotype groups. Bars represent mean ± SD. ^††^*p* < 0.01 *vs* control, ^##^*p* < 0.01 *vs RPGRIP1*^*ins*44/*ins*44^ (Dunn’s multiple comparisons test following a significant Kruskal–Wallis test).

To address the impact of *RPGRIP1* and *MAP9* variants on the primary cilium, we collected skin biopsies from control, *RPGRIP1* single affected, and *RPGRIP1*/*MAP9* double affected dogs and cultured CSFs. Following 48 h of serum starvation, the rate of ciliogenesis and the length of cilia were obtained from each CSF. Both the ciliogenesis rate and the axoneme length were comparable between *RPGRIP1* single affected and control CSFs. In contrast, the *RPGRIP1*/*MAP9* double affected CSFs had significantly reduced ciliogenesis and shorter axonemes compared to control CSFs (Figures 6A–C).

**FIGURE 7:**
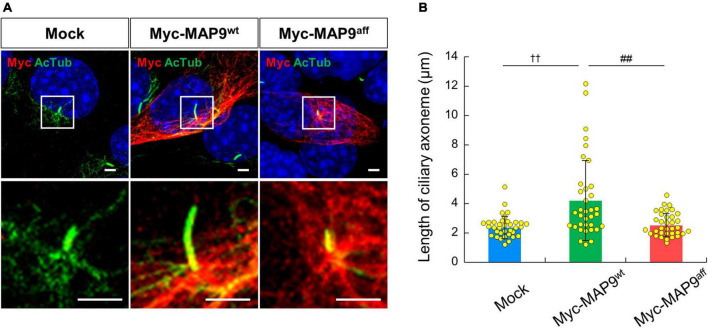
Overexpression of MAP9^wt^ leads to ciliary elongation in 661w cells. **(A)** ICC on 661W cells overexpressing canine derived MAP9 proteins, either wild type (MAP9^wt^) or affected (MAP9^aff^). Overexpressed myc-MAP9 and ciliary axoneme were visualized with anti-myc (red) and AcTub (green) antibodies, respectively. Nuclei were stained with Hoechst 33342 (blue). Scale bar, 5 μm. *Lower* panels are magnified images of the area outlined in the *Upper* panels. **(B)** Length of ciliary axoneme in control, myc-MAP9^wt^-overexpressing, and myc-MAP9^aff^-overexpressing cells (Number of wells = 3, Number of cells = 35). Bars represent mean ± SD. ^††^*p* < 0.01 *vs* control, ^##^*p* < 0.01 *vs* myc-MAP9^aff^ (Dunn’s multiple comparisons test following a significant Kruskal–Wallis test).

Finally, we observed the effect of overexpression of canine derived MAP9 proteins, either wild type (MAP9^wt^) or affected (MAP9^aff^) on ciliary length in 661W cells. Overexpressed MAP9 proteins, both MAP9^wt^ and MAP9^aff^, exhibited broad cytoplasmic expression but with increased signals corresponding to the base of primary cilium (Figure 7A). Interestingly, among the cells overexpressing MAP9^wt^, several cells with abnormally elongated ciliary axoneme were identified. Furthermore, the mean length of primary cilia in MAP9^wt^-overexpressing cells was significantly increased from that of untransfected cells. In contrast, there was no abnormal extension of ciliary axoneme in the MAP9^aff^-overexpressing cells (Figures 7A, B). Based on these results and other evidence showing a relationship between MAP9 and MT (Venoux et al., 2008; Tran et al., 2023), the MAP9^wt^ protein may play an important role in the formation and stabilization of ciliary axonemes. Meanwhile, the MAP9^aff^ protein has obviously different properties from MAP9^wt^ and may be deprived of critical roles in cilia stabilization. We also examined the expression pattern of endogenous RPGRIP1 in cells overexpressing MAP9 proteins and found no noticeable differences between cells receiving either treatment (Supplementary Figure 5).

## Discussion

Recently, through a long-term natural history study, we demonstrated the effect of a new modifier L3 that exacerbated cone dysfunction independent of *MAP9*^*aff*/*aff*^ in the canine oligogenic *RPGRIP1*-CRD model (Das et al., 2017; Ripolles-Garcia et al., 2023). As the role of MAP9 in photoreceptors remained unestablished, in the current study, we focused on the pathogenic impact of *MAP9^aff/aff^* alone on *RPGRIP1* mutants independent of the additional modifier L3. To capture the onset and early phenotype impacted by *MAP9*^*aff*/*aff*^ in *RPGRIP1* mutants, we focused specifically on the early stage of the disease, from shortly after birth to 17 months of age. Notably, the *RPGRIP1*/*MAP9* double affected retina showed almost complete loss of cone ERG and mildly reduced rod ERG as early as 1 month of age, and the subsequent degeneration of rod photoreceptors also occurred earlier and faster than in the *RPGRIP1* single affected retinas. While the undetectable cone ERG at 1 month of age in this double affected individual indicates a more severe presentation than the group of *RPGRIP1*/*MAP9* double affected dogs in our long-term study, the subsequent rapid decline in rod ERG highlights the role of *MAP9*^*aff*/*aff*^ in accelerating the pathology. Further, based on the cone-led phenotype in the dogs studies herein that are unaffected by L3, it can be concluded that L3 is not essential for the cone-led phenotype but rather exacerbates it. The initial impact on cones is also apparent by IHC of 17 months old retinas representing a more pronounced loss of cone OS compared to rod OS in the double affected. Therefore, it is suggested that *MAP9^aff/aff^* accelerates *RPGRIP1*^*ins*44/*ins*44^-induced CRD, leading to earlier disease onset, rapid progression, and more severe retinal degeneration.

Based on immunostaining of canine retinal sections and cultured cells, we found that MAP9 is prominently localized in the BB of photoreceptor neurosensory cilia and plays important roles in maintaining ciliary functions. Others have shown that endogenous MAP9 is strongly expressed in the centrosomal MT asters and the central spindle during mitosis in mammalian cells (Saffin et al., 2005; Venoux et al., 2008), indicating that it would be expressed in the BB in ciliated cells. On the other hand, the most recent publication using *C. elegans* claimed that MAPH-9, an ortholog of MAP9, is localized to the proximal region of the ciliary axoneme adjacent to the base of the cilia, rather than the ciliary BB (Tran et al., 2023). In addition, they also demonstrated that polyglutamylation on MT-axoneme is critical for the MAP9 localization to the ciliary axoneme, as suggested in other MAP proteins (Boucher et al., 1994; Bonnet et al., 2001). In this context, it is reasonable that endogenous MAP9 is localized in centrosomes and ciliary BBs, which undergo high levels of glutamylation in mammalian cells (Bobinnec et al., 1998). In the primary cilia of mammalian cells, it has been observed that there are distinct regions in the base of the ciliary axonemes that exhibit high levels of glutamylation (Kanamaru et al., 2022). Therefore, the extension of MAP9 signaling from the BB to the axoneme observed *in vitro* is expected to be dependent on the level of glutamylation of the ciliary axoneme.

We also assessed the localization of RPGRIP1 in the primary cilia, whose variants are the primary cause of this retinal degeneration model. In control canine retina, distinct RPGRIP1 signals were observed around the photoreceptor CC, consistent with previous reports in mouse retina (Patil et al., 2012). In contrast, RPGRIP1 signals in cultured cells did not colocalize with the ciliary axoneme but were detected rather surrounding the ciliary BB. This lack of RPGRIP1 localization in the transition zone in cultured cells has been reported by others (Wiegering et al., 2018), suggesting a cell-type and/or environment specific localization of RPGRIP1 in the cilia of photoreceptors. In addition, expression levels of *RPGRIP1* transcripts in photoreceptor cells were much higher compared to those in other human cell types. The photoreceptor-specific phenotype associated with *RPGRIP1* variants may be due to this photoreceptor-specific expression pattern of RPGRIP1.

The genomic and amino acid sequences of the *MAP9* modifier variants have been described in detail in the previous publication (Forman et al., 2016). In this variant, there is an approximately 22 kb deletion after intron 10 of *MAP9*, while the difference in amino acid sequence between MAP9^wt^ and MAP9^aff^ is very small due to the joining of a subsequent *MAP9*-like pseudogene. Moreover, the amino acid substitutions and deletions in MAP9^aff^ are mostly concentrated around the c-terminus after amino acid residue 489, which is consistent with the MAP domain of MAP9 (Venoux et al., 2008). Based on these reports, we expected that MAP9^aff^ might be defective in functions related to stabilization of the ciliary MT-axoneme of photoreceptors. Indeed, we have demonstrated that the *MAP9* variant affects the length of the ciliary axoneme in a cultured cell system. A study by others has suggested that loss of *MAPH-9* causes ultrastructural microtubule doublet defects in the ciliary axoneme of *C. elegans* (Tran et al., 2023). In addition, it has been reported that other MAPs also promote MT stabilization through interaction with tubulins (Conkar and Firat-Karalar, 2021; Odabasi et al., 2023). Thus, it is suggested that MAP9, a member of the MAPs, may contribute to the structural integrity of photoreceptor cilia through stabilization of the ciliary axonemes. Besides MT stabilization, MAP9 has also been associated with motor protein-mediated ciliary trafficking (Monroy et al., 2020), indicating that further investigation of the molecular function of MAP9 in primary cilium is warranted.

What remains to be investigated includes the direct interaction between RPGRIP1 and MAP9 of each the WT and the variant form. In addition, disease exacerbation caused by the *MAP9* variant is only observed in the presence of *RPGRIP1*^*ins*44/*ins*44^, and *MAP9^aff/aff^* itself has not been associated with a phenotype. Therefore, further studies using MAP9 knock-out models, etc. are needed to clarify the effects of the complete loss of MAP9 on photoreceptor cilia as well as primary cilia in other cell types. Another limitation of this study is that insufficient biological replicates due to the utilization of a canine model. While the comparisons between the littermates clearly indicate that *MAP9^aff/aff^* causes earlier and more severe *RPGRIP1*-associated retinal degeneration, accumulation of larger sample sizes will be needed to provide a more general conclusion based on statistical examination.

In summary, through phenotypic analysis of the canine *RPGRIP1*-CRD model, we demonstrated that MAP9 plays an important role in the formation and functional regulation of primary cilia. The MAP9^aff^ protein may be deprived of critical roles in cilia organization and maintenance resulting in altered cilia structure and function giving rise to early onset and accelerated disease progression observed in *RPGRIP1*-associated CRD among the double homozygotes. Our findings suggest that the role of MAP9 in normal retinal function warrants further investigation, including functional assessment of MAP9 protein as well as screening for *MAP9* variants in IRDs patients whose underlying genetic variants have not been accounted for, particularly if there are indications of ciliopathy.

## Data availability statement

The datasets presented in this study can be found in online repositories. The names of the repository/repositories and accession number(s) can be found in the article/Supplementary material.

## Ethics statement

This research was conducted in strict accordance with the recommendations of the NIH [National Research Council (US) Committee, 2011] and in full compliance with the US Department of Agriculture’s Animal Welfare Act, Animal Welfare Regulations, and the ARVO Statement for the Use of Animals in Ophthalmic and Vision Research. The protocols were approved by the Institutional Animal Care and Use Committee (IACUC) of the University of Pennsylvania (IACUC, Protocol# 804956).

## Author contributions

KT and KM contributed to the conception or design of the work. KT, JK, YS, GA, and KM contributed to the acquisition, analysis, or interpretation of data for this manuscript. KT wrote the drafted manuscript. GA and KM reviewed and edited the manuscript. All authors reviewed the manuscript, approved submission of this manuscript and take full responsibility for the manuscript.

## Footnotes

1 https://www.proteinatlas.org/; accessed 5/10/23.

## Supplementary material

Supplementary Figure 1 IHC on late-stage *RPGRIP1*-associated CRD retinas. IHC on 5 years and 9 months old *RPGRIP1*^*ins*44/*ins*44^ retina **(A,C)** and 6 years old *RPGRIP1*^*ins*44/*ins*44^ MAP9*^aff/aff^* retina **(B,D)** with anti-hCAR (green) and rhodopsin (red) antibodies. The *upper* and *lower* panels show superior and inferior retina, respectively. Nuclei were stained with Hoechst 33342 (blue). INL, inner nuclear layer; IS + OS, inner segment + outer segment; ONL, outer nuclear layer. Scale bar, 10 μm.

Supplementary Figure 2 Higher expression of *MAP9* and *RPGRIP1* transcripts in human photoreceptors. Meta-analysis on normalized human single cell transcriptomic dataset from the HPA database. Expression levels of *MAP9* **(A)** and *RPGRIP1* **(B)** transcripts between different cell types were compared using the dataset derived from this database. *MAP9* and *RPGRIP1* transcripts were abundantly expressed in both rod and cone photoreceptors.

Supplementary Figure 3 Validation of MAP9 signal patterns using alternative anti-MAP9 antibody. To validate the reliability of MAP9 protein localization patterns, immunostaining with an alternative anti-MAP9 antibody (Novus Biologicals, Cat# NBP1-90842) was conducted. **(A,B)** Normal canine retinas are co-immunolabeled with AcTub (green) or γ-tubulin (gray) along with either MAP9 (red). The endogenous MAP9 protein signal was comparable to that in Figures 4A, B. **(C)** ICC on 661W cells overexpressing MAP9^wt^ or MAP9^aff^. The anti-MAP9 antibody had similar labeling patterns to the anti-Myc antibody for both MAP9^wt^ and MAP9^aff^. Nuclei were visualized with Hoechst 33342 (blue). CC, connecting cilium; IS, inner segment; ONL, outer nuclear layer. Scale bar, 10 μm.

Supplementary Figure 4 Multiple sequence alignment for MAP9 proteins. Comparison of amino acid sequences between canine wild type, affected, and human MAP9 proteins. The immunogens for the two anti-MAP9 antibodies used in present study were highlighted in red and green. Variants in MAP9^aff^ were highlighted in blue.

Supplementary Figure 5 Expression pattern of endogenous RPGRIP1 in myc-MAP9 overexpressed 661W cells. ICC on 661W cells overexpressing canine-derived MAP9 proteins, either MAP9^wt^ or MAP9^aff^. Overexpressed myc-MAP9 and endogenous RPGRIP1 were visualized with anti-myc (green) and RPGRIP1 (red) antibodies. No noticeable differences in the expression pattern of RPGRIP1 were found between cells receiving either treatment. Nuclei were stained with Hoechst 33342 (blue). Scale bar, 5 μm.

## References

[B1] Aguirre G. D. Cideciyan A. V. Dufour V. L. Ripolles-García A. Sudharsan R. Swider M. (2021). Gene therapy reforms photoreceptor structure and restores vision in NPHP5-associated Leber congenital amaurosis. *Mol. Ther.* 29 2456–2468. 10.1016/j.ymthe.2021.03.021 33781914 PMC8353203

[B2] Barnes C. L. Malhotra H. Calvert P. D. (2021). Compartmentalization of photoreceptor sensory cilia. *Front. Cell Dev. Biol.* 9:636737. 10.3389/fcell.2021.636737 33614665 PMC7889997

[B3] Beryozkin A. Aweidah H. Carrero Valenzuela R. D. Berman M. Iguzquiza O. Cremers F. P. M. (2021). Retinal degeneration associated with RPGRIP1: A review of natural history, mutation spectrum, and genotype-phenotype correlation in 228 patients. *Front. cell Dev. Biol.* 9:746781. 10.3389/fcell.2021.746781 34722527 PMC8551679

[B4] Bobinnec Y. Moudjou M. Fouquet J. P. Desbruyères E. Eddé B. Bornens M. (1998). Glutamylation of centriole and cytoplasmic tubulin in proliferating non-neuronal cells. *Cell Motil. Cytoskeleton* 39 223–232. 10.1002/(SICI)1097-0169199839:3<223::AID-CM5<3.0.CO;2-5 9519903

[B5] Bonnet C. Boucher D. Lazereg S. Pedrotti B. Islam K. Denoulet P. (2001). Differential binding regulation of microtubule-associated proteins MAP1A, MAP1B, and MAP2 by tubulin polyglutamylation. *J. Biol. Chem.* 276 12839–12848. 10.1074/jbc.M011380200 11278895

[B6] Booij J. C. Florijn R. J. ten Brink J. B. Loves W. Meire F. van Schooneveld M. J. (2005). Identification of mutations in the AIPL1, CRB1, GUCY2D, RPE65, and RPGRIP1 genes in patients with juvenile retinitis pigmentosa. *J. Med. Genet.* 42:e67. 10.1136/jmg.2005.035121 16272259 PMC1735944

[B7] Boucher D. Larcher J. C. Gros F. Denoulet P. (1994). Polyglutamylation of tubulin as a progressive regulator of in vitro interactions between the microtubule-associated protein tau and tubulin. *Biochemistry* 33 12471–12477. 10.1021/bi00207a014 7522559

[B8] Chang B. Khanna H. Hawes N. Jimeno D. He S. Lillo C. (2006). In-frame deletion in a novel centrosomal/ciliary protein CEP290/NPHP6 perturbs its interaction with RPGR and results in early-onset retinal degeneration in the rd16 mouse. *Hum. Mol. Genet.* 15 1847–1857. 10.1093/hmg/ddl107 16632484 PMC1592550

[B9] Conkar D. Firat-Karalar E. N. (2021). Microtubule-associated proteins and emerging links to primary cilium structure, assembly, maintenance, and disassembly. *FEBS J.* 288 786–798. 10.1111/febs.15473 32627332

[B10] Das R. G. Marinho F. P. Iwabe S. Santana E. McDaid K. S. Aguirre G. D. (2017). Variabilities in retinal function and structure in a canine model of cone-rod dystrophy associated with RPGRIP1 support multigenic etiology. *Sci. Rep.* 7:12823. 10.1038/s41598-017-13112-w 28993665 PMC5634483

[B11] Dryja T. P. Adams S. M. Grimsby J. L. McGee T. L. Hong D. H. Li T. (2001). Null RPGRIP1 alleles in patients with Leber congenital amaurosis. *Am. J. Hum. Genet.* 68 1295–1298. 10.1086/320113 11283794 PMC1226111

[B12] Eandi C. M. Charles Messance H. Augustin S. Dominguez E. Lavalette S. Forster V. (2016). Subretinal mononuclear phagocytes induce cone segment loss via IL-1β. *Elife* 5:e16490. 10.7554/eLife.16490 27438413 PMC4969036

[B13] Eblimit A. Nguyen T.-M. T. Chen Y. Esteve-Rudd J. Zhong H. Letteboer S. (2015). Spata7 is a retinal ciliopathy gene critical for correct RPGRIP1 localization and protein trafficking in the retina. *Hum. Mol. Genet.* 24 1584–1601. 10.1093/hmg/ddu573 25398945 PMC4351378

[B14] Fontenille L. Rouquier S. Lutfalla G. Giorgi D. (2014). Microtubule-associated protein 9 (Map9/Asap) is required for the early steps of zebrafish development. *Cell Cycle* 13 1101–1114. 10.4161/cc.27944 24553125 PMC4013161

[B15] Forman O. P. Hitti R. J. Boursnell M. Miyadera K. Sargan D. Mellersh C. (2016). Canine genome assembly correction facilitates identification of a MAP9 deletion as a potential age of onset modifier for RPGRIP1-associated canine retinal degeneration. *Mamm. Genome* 27 237–245. 10.1007/s00335-016-9627-x 27017229

[B16] Hameed A. Abid A. Aziz A. Ismail M. Mehdi S. Q. Khaliq S. (2003). Evidence of RPGRIP1 gene mutations associated with recessive cone-rod dystrophy. *J. Med. Genet.* 40 616–619. 10.1136/jmg.40.8.616 12920076 PMC1735563

[B17] Hofmann K. P. Lamb T. D. (2023). Rhodopsin, light-sensor of vision. *Prog. Retin. Eye Res.* 93:101116. 10.1016/j.preteyeres.2022.101116 36273969

[B18] Hong D. H. Yue G. Adamian M. Li T. (2001). Retinitis pigmentosa GTPase regulator (RPGRr)-interacting protein is stably associated with the photoreceptor ciliary axoneme and anchors RPGR to the connecting cilium. *J. Biol. Chem.* 276 12091–12099. 10.1074/jbc.M009351200 11104772

[B19] Jensen V. L. Carter S. Sanders A. A. Li C. Kennedy J. Timbers T. A. (2016). Whole-organism developmental expression profiling identifies RAB-28 as a novel ciliary GTPase associated with the bbsome and intraflagellar transport. *PLoS Genet.* 12:e1006469. 10.1371/journal.pgen.1006469 27930654 PMC5145144

[B20] Kanamaru T. Neuner A. Kurtulmus B. Pereira G. (2022). Balancing the length of the distal tip by septins is key for stability and signalling function of primary cilia. *EMBO J.* 41:e108843. 10.15252/embj.2021108843 34981518 PMC8724769

[B21] Kuznetsova T. Iwabe S. Boesze-Battaglia K. Pearce-Kelling S. Chang-Min Y. McDaid K. (2012). Exclusion of RPGRIP1 ins44 from primary causal association with early-onset cone-rod dystrophy in dogs. *Invest. Ophthalmol. Vis. Sci.* 53 5486–5501. 10.1167/iovs.12-10178 22807295 PMC3422103

[B22] Liu W. Xiao H. Wu S. Liu H. Luo B. (2018). MAP9 single nucleotide polymorphism rs1058992 is a risk of EBV-associated gastric carcinoma in Chinese population. *Acta Virol.* 62 435–440. 10.4149/av_2018_412 30472874

[B23] Magescas J. Eskinazi S. Tran M. V. Feldman J. L. (2021). Centriole-less pericentriolar material serves as a microtubule organizing center at the base of *C. elegans* sensory cilia. *Curr. Biol.* 31 2410–2417.e6. 10.1016/j.cub.2021.03.022 33798428 PMC8277230

[B24] Mao Y. Long Y. Liu B. Cao Q. Li Y. Li S. (2021). Ocular characteristics of patients with leber congenital amaurosis 6 caused by pathogenic RPGRIP1 gene variation in a Chinese cohort. *J. Ophthalmol.* 2021:9966427. 10.1155/2021/9966427 34796026 PMC8595035

[B25] Mellersh C. S. Boursnell M. E. G. Pettitt L. Ryder E. J. Holmes N. G. Grafham D. (2006). Canine RPGRIP1 mutation establishes cone-rod dystrophy in miniature longhaired dachshunds as a homologue of human leber congenital amaurosis. *Genomics* 88 293–301. 10.1016/j.ygeno.2006.05.004 16806805

[B26] Miyadera K. Kato K. Aguirre-Hernández J. Tokuriki T. Morimoto K. Busse C. (2009). Phenotypic variation and genotype-phenotype discordance in canine cone-rod dystrophy with an RPGRIP1 mutation. *Mol. Vis.* 15 2287–2305. 19936303 PMC2779058

[B27] Miyadera K. Acland G. M. Aguirre G. D. (2012a). Genetic and phenotypic variations of inherited retinal diseases in dogs: The power of within- and across-breed studies. *Mamm. Genome* 23 40–61. 10.1007/s00335-011-9361-3 22065099 PMC3942498

[B28] Miyadera K. Kato K. Boursnell M. Mellersh C. S. Sargan D. R. (2012b). Genome-wide association study in RPGRIP1(-/-) dogs identifies a modifier locus that determines the onset of retinal degeneration. *Mamm. Genome* 23 212–223. 10.1007/s00335-011-9384-9 22193413 PMC3947618

[B29] Monroy B. Y. Tan T. C. Oclaman J. M. Han J. S. Simó S. Niwa S. (2020). A combinatorial MAP code dictates polarized microtubule transport. *Dev. Cell* 53 60–72.e4. 10.1016/j.devcel.2020.01.029 32109385 PMC7181406

[B30] National Research Council (US) Committee, (2011). *Guide for the care and use of laboratory animals.* Washington, DC: National Academies Press. 10.17226/12910

[B31] Odabasi E. Conkar D. Deretic J. Batman U. Frikstad K.-A. M. Patzke S. (2023). CCDC66 regulates primary cilium length and signaling via interactions with transition zone and axonemal proteins. *J. Cell Sci.* 136:jcs260327. 10.1242/jcs.260327 36606424

[B32] Patil H. Tserentsoodol N. Saha A. Hao Y. Webb M. Ferreira P. A. (2012). Selective loss of RPGRIP1-dependent ciliary targeting of NPHP4, RPGR and SDCCAG8 underlies the degeneration of photoreceptor neurons. *Cell Death Dis.* 3:e355. 10.1038/cddis.2012.96 22825473 PMC3406595

[B33] Ripolles-Garcia A. Murgiano L. Ziolkowska N. Marinho F. P. Roszak K. Iffrig S. (2023). Natural disease history of a canine model of oligogenic RPGRIP1-cone-rod dystrophy establishes variable effects of previously and newly mapped modifier loci. *Hum. Mol. Genet.* 32 2139–51. 10.1093/hmg/ddad046 36951959 PMC10281748

[B34] Roepman R. Letteboer S. J. F. Arts H. H. van Beersum S. E. C. Lu X. Krieger E. (2005). Interaction of nephrocystin-4 and RPGRIP1 is disrupted by nephronophthisis or leber congenital amaurosis-associated mutations. *Proc. Natl. Acad. Sci. U.S.A.* 102 18520–18525. 10.1073/pnas.0505774102 16339905 PMC1317916

[B35] Saffin J.-M. Venoux M. Prigent C. Espeut J. Poulat F. Giorgi D. (2005). ASAP, a human microtubule-associated protein required for bipolar spindle assembly and cytokinesis. *Proc. Natl. Acad. Sci. U.S.A.* 102 11302–11307. 10.1073/pnas.0500964102 16049101 PMC1183541

[B36] Sánchez-Bellver L. Toulis V. Marfany G. (2021). On the wrong track: Alterations of ciliary transport in inherited retinal dystrophies. *Front. cell Dev. Biol.* 9:623734. 10.3389/fcell.2021.623734 33748110 PMC7973215

[B37] Tan E. Ding X.-Q. Saadi A. Agarwal N. Naash M. I. Al-Ubaidi M. R. (2004). Expression of cone-photoreceptor-specific antigens in a cell line derived from retinal tumors in transgenic mice. *Invest. Ophthalmol. Vis. Sci.* 45 764–768. 10.1167/iovs.03-1114 14985288 PMC2937568

[B38] Tran M. V. Ferguson J. W. Cote L. E. Khuntsariya D. Fetter R. D. Wang J. T. (2023). MAP9/MAPH-9 supports axonemal microtubule doublets and modulates motor movement. *bioRxiv* [Preprint]. 10.1101/2023.02.23.529616 38159567 PMC11385174

[B39] Venoux M. Delmouly K. Milhavet O. Vidal-Eychenié S. Giorgi D. Rouquier S. (2008). Gene organization, evolution and expression of the microtubule-associated protein ASAP (MAP9). *BMC Genomics* 9:406. 10.1186/1471-2164-9-406 18782428 PMC2551623

[B40] Wang S. Huang J. Li C. Zhao L. Wong C. C. Zhai J. (2020). MAP9 loss triggers chromosomal instability, initiates colorectal tumorigenesis, and is associated with poor survival of patients with colorectal cancer. *Clin. Cancer Res.* 26 746–757. 10.1158/1078-0432.CCR-19-1611 31662330

[B41] Wensel T. G. Potter V. L. Moye A. Zhang Z. Robichaux M. A. (2021). Structure and dynamics of photoreceptor sensory cilia. *Pflugers Arch.* 473 1517–1537. 10.1007/s00424-021-02564-9 34050409 PMC11216635

[B42] Wheway G. Nazlamova L. Turner D. Cross S. (2019). 661W photoreceptor cell line as a cell model for studying retinal ciliopathies. *Front. Genet.* 10:308. 10.3389/fgene.2019.00308 31024622 PMC6459963

[B43] Wiegering A. Dildrop R. Kalfhues L. Spychala A. Kuschel S. Lier J. M. (2018). Cell type-specific regulation of ciliary transition zone assembly in vertebrates. *EMBO J.* 37:e97791. 10.15252/embj.201797791 29650680 PMC5978567

[B44] Zhang J. Huang J.-Z. Zhang Y.-Q. Zhang X. Zhao L.-Y. Li C.-G. (2020). Microtubule associated protein 9 inhibits liver tumorigenesis by suppressing ERCC3. *EBioMedicine* 53:102701. 10.1016/j.ebiom.2020.102701 32151798 PMC7063135

