# Supplementary figures and images for: Molecular characterization of MAP9 in the photoreceptor sensory cilia as a modifier in canine RPGRIP1-associated cone-rod dystrophy
Source: Front Cell Neurosci. 2023 Aug 15;17:1226603. doi: 10.3389/fncel.2023.1226603 (PMC10464610; doi:10.3389/fncel.2023.1226603)

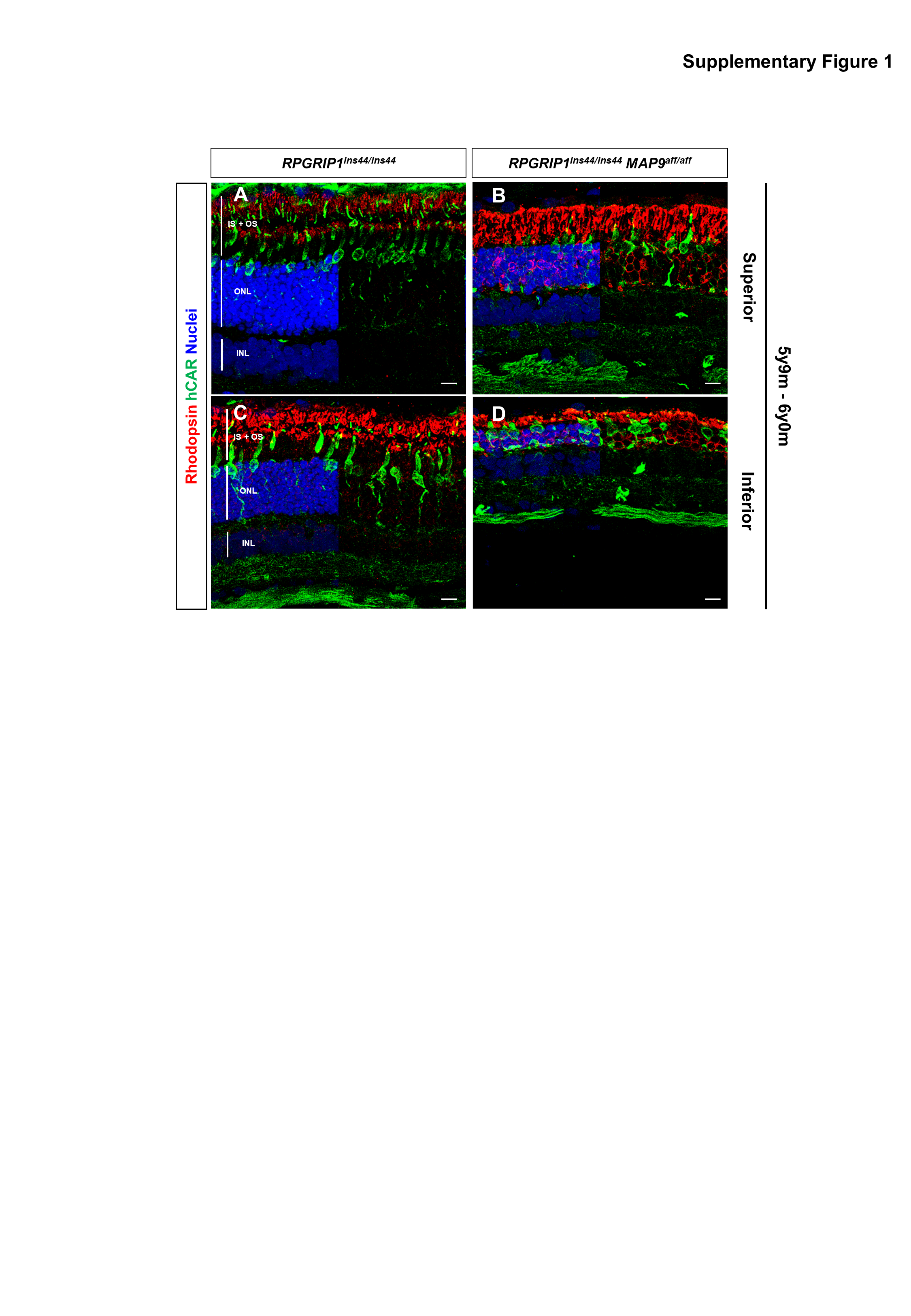

Supplement: Supplementary Figure 1 — IHC on late-stage RPGRIP1-associated CRD retinas. IHC on 5 years and 9 months old RPGRIP1ins44/ins44 retina (A,C) and 6 years old RPGRIP1ins44/ins44 MAP9aff/aff retina (B,D) with anti-hCAR (green) and rhodopsin (red) antibodies. The upper and lower panels show superior and inferior retina, respectively. Nuclei were stained with Hoechst 33342 (blue). INL, inner nuclear layer; IS + OS, inner segment + outer segment; ONL, outer nuclear layer. Scale bar, 10 μm. [file Image_1.TIF]

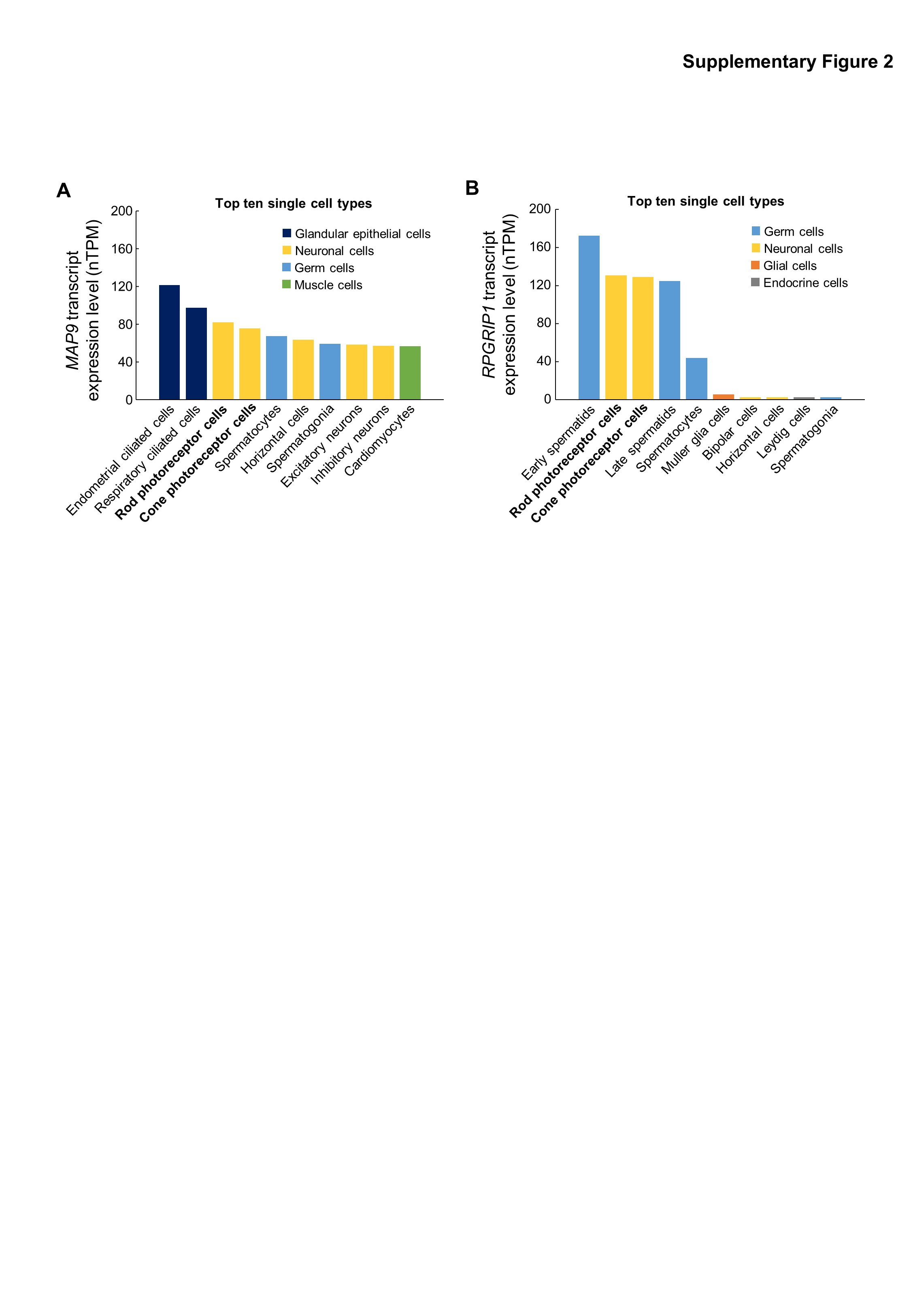

Supplement: Supplementary Figure 2 — Higher expression of MAP9 and RPGRIP1 transcripts in human photoreceptors. Meta-analysis on normalized human single cell transcriptomic dataset from the HPA database. Expression levels of MAP9 (A) and RPGRIP1 (B) transcripts between different cell types were compared using the dataset derived from this database. MAP9 and RPGRIP1 transcripts were abundantly expressed in both rod and cone photoreceptors. [file Image_2.TIF]

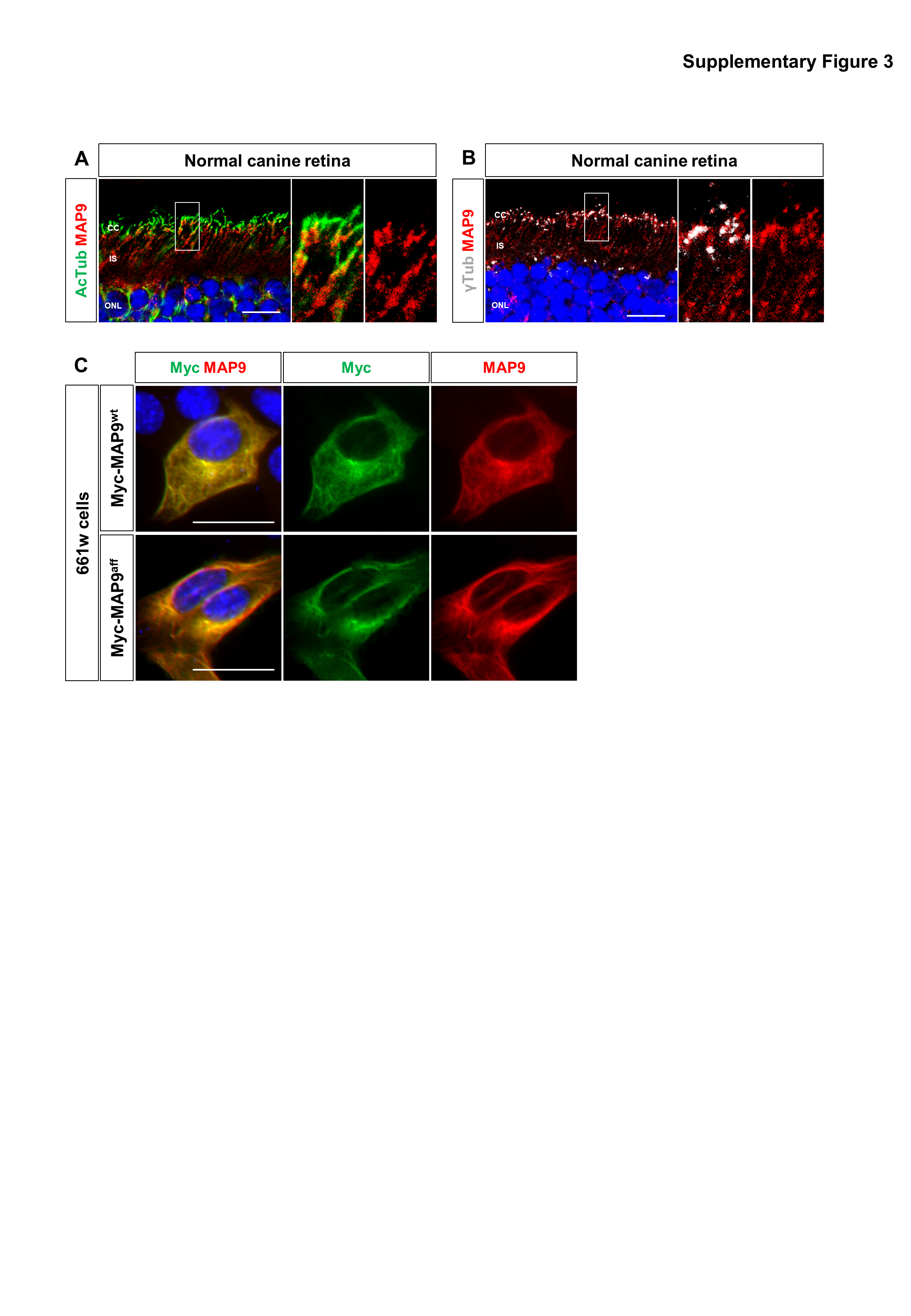

Supplement: Supplementary Figure 3 — Validation of MAP9 signal patterns using alternative anti-MAP9 antibody. To validate the reliability of MAP9 protein localization patterns, immunostaining with an alternative anti-MAP9 antibody (Novus Biologicals, Cat# NBP1-90842) was conducted. (A,B) Normal canine retinas are co-immunolabeled with AcTub (green) or γ-tubulin (gray) along with either MAP9 (red). The endogenous MAP9 protein signal was comparable to that in Figures 4A, B. (C) ICC on 661W cells overexpressing MAP9wt or MAP9aff. The anti-MAP9 antibody had similar labeling patterns to the anti-Myc antibody for both MAP9wt and MAP9aff. Nuclei were visualized with Hoechst 33342 (blue). CC, connecting cilium; IS, inner segment; ONL, outer nuclear layer. Scale bar, 10 μm. [file Image_3.TIF]

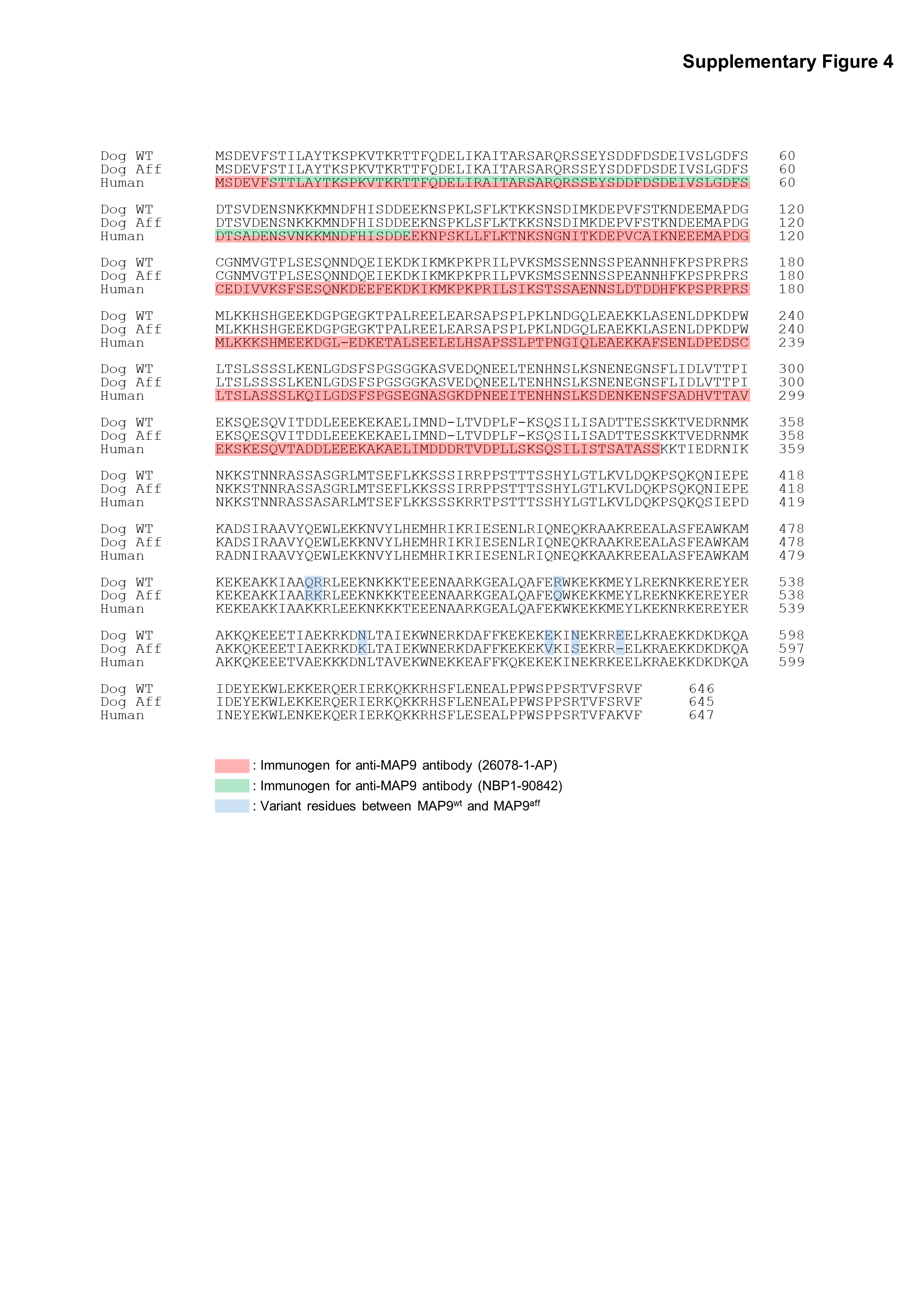

Supplement: Supplementary Figure 4 — Multiple sequence alignment for MAP9 proteins. Comparison of amino acid sequences between canine wild type, affected, and human MAP9 proteins. The immunogens for the two anti-MAP9 antibodies used in present study were highlighted in red and green. Variants in MAP9aff were highlighted in blue. [file Image_4.TIF]

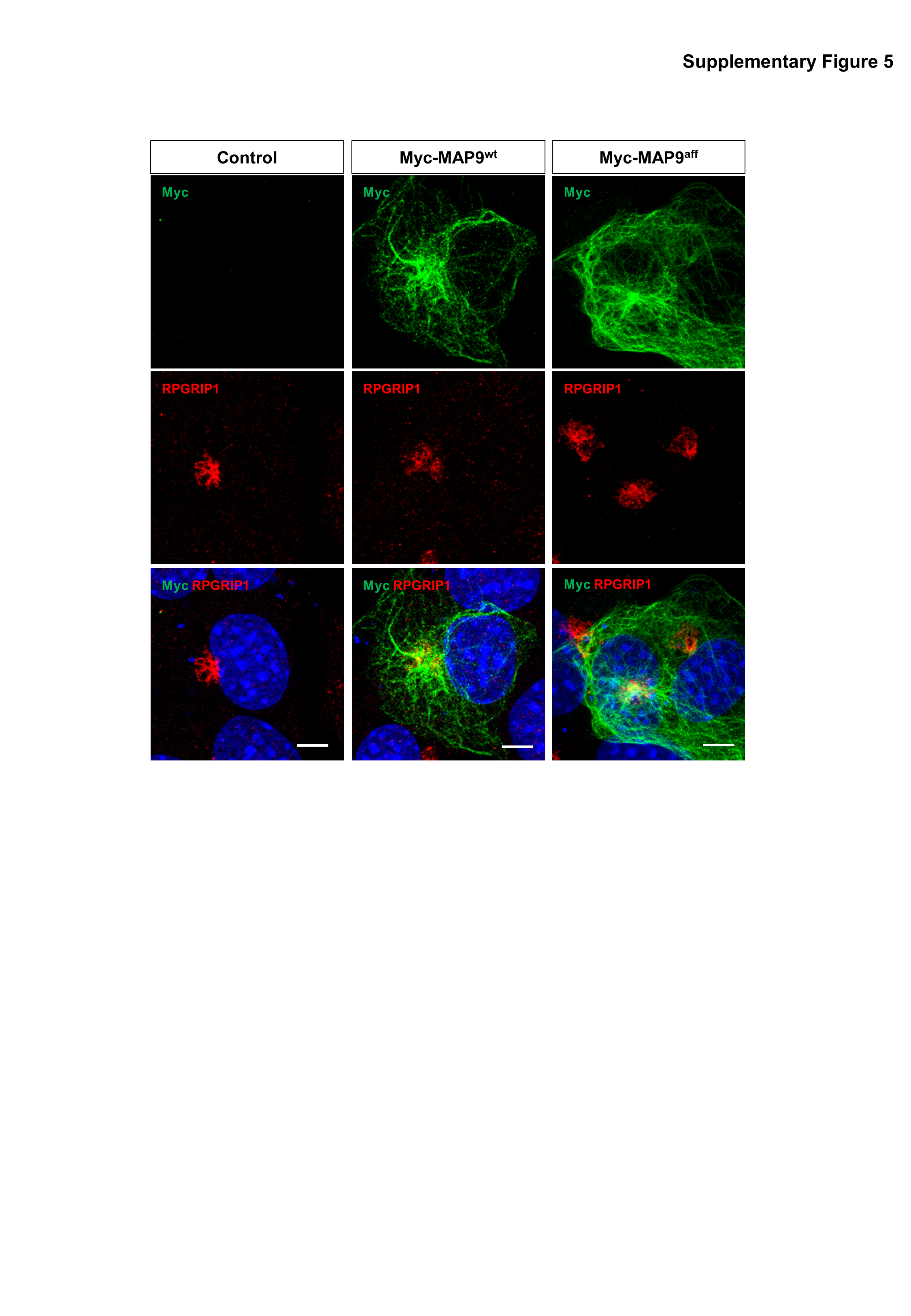

Supplement: Supplementary Figure 5 — Expression pattern of endogenous RPGRIP1 in myc-MAP9 overexpressed 661W cells. ICC on 661W cells overexpressing canine-derived MAP9 proteins, either MAP9wt or MAP9aff. Overexpressed myc-MAP9 and endogenous RPGRIP1 were visualized with anti-myc (green) and RPGRIP1 (red) antibodies. No noticeable differences in the expression pattern of RPGRIP1 were found between cells receiving either treatment. Nuclei were stained with Hoechst 33342 (blue). Scale bar, 5 μm. [file Image_5.TIF]
